# Development of reed-based cellulose aerogel: a sustainable solution for crude oil spill clean-up

**DOI:** 10.1098/rsos.241207

**Published:** 2025-01-15

**Authors:** Huong Le Thi Thanh, An Tran Nguyen Minh, Hoang Tran Huu

**Affiliations:** ^1^ Hydro-Meteorology and Climate Change Faculty, Ho Chi Minh University of Natural Resources and Environment (HCMUNRE), Ho Chi Minh 72100, Vietnam; ^2^ Faculty of Chemical Engineering, Industrial University of Ho Chi Minh City (IUH), Ho Chi Minh 71420, Vietnam

**Keywords:** aerogel, reed, kraft pulping, cellulose, methyltrimethoxysilane, crude oil absorption

## Abstract

This study focused on fabricating a cellulose aerogel for oil spill clean-up, using common reed (*Phragmites australis*) as the cellulose source. The process involved isolating cellulose from reed via traditional Kraft pulping, considering the effects of key factors on the isolated cellulose content. After a two-stage HP bleaching sequence, the highest cellulose content achieved was 27.2%, with 80% ISO brightness and 1% ash content under mild Kraft pulping conditions of 30% sulfidity, 20% active alkali (AA), sustained cooking at 165°C for 3 h, and a liquor-to-reed ratio of 8 : 1. Subsequently, reed-based cellulose aerogel was fabricated via a freeze-drying method using an eco-friendly NaOH/poly(ethylene glycol) aqueous solvent system, which was then modified with methyltrimethoxysilane. The resulting aerogel exhibited remarkable characteristics, including a low density of 0.04 g cm^−3^, high porosity of 96%, high hydrophobicity with a water contact angle (WAC) of 141°, and a superior crude oil adsorption capacity of 35 g g^−1^. Comprehensive characterizations of the fabricated materials, including scanning electron microscopy, Fourier transform infrared spectroscopy, thermogravimetric analysis/differential scanning calorimetry, and WAC measurements, were evaluated. This interdisciplinary study explores the commercial promise of reed-based cellulose aerogel as a sustainable solution for oil spill clean-up efforts.

## 1. Introduction

Collecting and cleaning oil spills at sea is an important environmental concern because of the negative impacts of spilled oil on marine ecosystems, vegetation and human health. In the context of climate change, it is imperative to explore innovative methods and new materials that can effectively absorb spilled oil while being cost-effective, reusable and derived from renewable sources. Aerogels are known for their unique properties such as high porosity, low density, large surface area, excellent thermal insulation and modifiable surface chemistry [1,2]. Previous research has highlighted the effectiveness of aerogels, especially those derived from various cellulose sources, as materials for absorbing spilled oil [1–4]. Researchers and industrial representatives are increasingly interested in cellulose aerogels derived from non-wood sources because of their potential as alternative materials, as well as their availability, affordability, biodegradability, degradability, recyclability and easy surface modification. The raw materials to isolate cellulose feedstocks include waste paper [5–7], cotton [8], a wide variety of crop straws [3,9–13] and various plants [12,14,15]. Different types of aerogels based on cellulose, nanocellulose (NC), and microcrystalline cellulose (MCC) derived from plant sources have been investigated, showing promising results for oil absorption. Inheriting the properties of cellulose, NC, in the forms of cellulose nanocrystals and cellulose nanofibrils, also exhibits unique characteristics such as nanoscale size, distinctive morphology and a large surface area, enabling it to offer superior performance in oil spill clean-up. However, significant economic and environmental challenges remain, including high production costs and the need for a complex procedure, advanced technology and equipment to break down cellulose fibres into nanoscale particles and to control NC quality, especially when organic solvents are required. These barriers prevent NC from fully reaching its potential [16–21]. MCC is derived from cellulose through acid hydrolysis using mineral acids, enzymes or microorganisms. However, the properties of MCC, including crystallinity, moisture content, molecular weight, surface area, porosity and thermal resistance, vary depending on the source of the raw material [20,22,23]. Compared to chemically untreated virgin cellulose, MCC is more expensive and requires additional stages in the production process. Typically, purified MCC is directly used as a feedstock in most studies focused on aerogel preparation from MCC [20,24]. Cellulose is more readily available, eco-friendly, cost-effective and simpler to isolation process. This makes cellulose a highly viable material for commercial aerogel production [1,3,4]. The non-wood cellulose materials used in current research for aerogel preparation are typically purified cellulose or obtained through laboratory methods such as solvent extraction, enzyme treatment, acid hydrolysis or a combination of ultrasonication and chemical treatment. Therefore, more economical, environmentally benign and efficient methods for the practical production of non-wood cellulose-based aerogels need increased attention and development.

Freeze-drying is one of the most effective methods for preparing cellulose aerogels. During this process, all moisture is removed through sublimation, preventing the collapse of the aerogel structure and leaving a network of pure solid cellulose fibres [3,9]. However, dissolving cellulose in common aqueous or organic solvents for freeze-drying can be challenging because of the strong inter- and intramolecular hydrogen bonding, and close chain packing facilitated by numerous hydrogen bonds. Lifeng Yan introduced an aqueous solvent system composed of a mixture of NaOH and poly(ethylene glycol) (PEG), described as an eco-benign, low-cost and efficient solution for dissolving cellulose [11,25,26]. Due to the inherent hydrophilicity of cellulose, further modification of the cellulose aerogel is necessary to achieve hydrophobicity, a critical property for oil sorbents [14,25,27]. Methyltrimethoxysilane (MTMS) is commonly used for this purpose due to its straightforward modification process and cost-effectiveness [1,27,28].

Over the past decade, the common reed (*Phragmites australis*), also known as reed, has become a valuable non-wood resource in the pulping and papermaking industries. Existing literature reports reed cellulose contents ranging from 33 to 59%, which are influenced by environmental factors [29–31]. In addition to its status as an invasive large perennial plant with global distribution, reed offers advantages such as availability, cost-effectiveness, biodegradability, renewability and recyclability. Additionally, reed-based cellulose exhibits some favourable properties such as excellent drainability, transparency, good mechanical strength and low silicon content (≤1.12 g l^−1^) compared to cellulose from other sources [29,31,32]. Despite these promising attributes, there remains limited information about using cellulose isolated from reed as a feedstock for aerogel fabrication. The report by Jiao and colleagues on the use of natural reed powder as a feedstock for the development of nanofibrillated cellulose aerogels is one of the relatively rare studies found. However, this process presents challenges for green technology due to its complicated stages for cellulose extraction and the use of a mixture of organic solvents, including benzene and ethanol [33].

In this study, cellulose aerogels (RCA) were fabricated via a freeze-drying method using cellulose isolated from the common reed (RC) as the raw material and an eco-friendly NaOH/PEG aqueous solvent system. The Kraft pulping method was applied for cellulose isolation because it is a conventional pulping technique widely used in the pulp and paper industries and can be applied to a wide range of raw materials. Moreover, it produces high-quality cellulose with good physical–mechanical properties and improved bleachability, especially as it efficiently recovers chemicals [34,35]. The effects of main pulping variables on the isolated cellulose content were explored, such as the applied charge of AA, sodium sulfide, cooking time and liquid-to-reed (L : Q) ratio [36–38]. The fabricated aerogel was modified with MTMS (referred to as RCA_MTMS), followed by evaluating its adsorption capacity for origin crude oil and oil/water mixtures. Each experiment was replicated, and average results were calculated. The morphologies, structures and properties of RC, RCA and RCA_MTMS were characterized and compared using various techniques, including brightness, ash content, scanning electron microscopy (SEM), Fourier transform infrared spectroscopy (FTIR), thermogravimetric analysis (TGA), Brunauer–Emmett–Teller (BET) surface area analysis and water contact angle (WCA) measurements.

## 2. Material and methods

### 2.1. Materials

Reeds were collected from District 2 near the Saigon River in Ho Chi Minh City. The reed stems, node-free, were manually cut into approximately 3−5 mm pieces. These were then washed with water, oven-dried at 40°C and stored until a constant weight was achieved. Before pulping, reed chips underwent a 24 h impregnation with the pulping liquor to increase the isolated cellulose content and reduce the pulping time. All chemicals of analytical grade were purchased from Chinese suppliers, including NaOH, HCl, Na_2_S.9H_2_O, NaClO and H_2_O_2_, and were used without further purification. PEG with a molecular weight of 6000 was obtained from India, and MTMS (99.8%) was supplied by Sigma. All solutions were prepared in distilled water. The solvent used was an aqueous solution containing a mixture of 5% PEG and 5% NaOH.

### 2.2. Isolation of cellulose from reed

Pulping was performed following the traditional Kraft cooking technique, with a few minor modifications [39–42]. The pulping of 0.5 kg oven-dried reed chips was conducted under constant conditions of temperature (165°C) and pressure (0.7 MPa) in a 15 l laboratory-scale batch digester. This electrically heated, rotating digester was equipped with an automatic processor to control preset parameters such as cooking time, pressure and temperature. The effect of pulping variables was examined using the one-factor-at-a-time approach. Based on experimental and reported results, four investigated key variables of Kraft pulping were adjusted within the following ranges: sulfidity (10–35%), AA as Na_2_O (AA, 10–25%), L : Q ratio (6 : 1−12 : 1) and cooking time (2.0−3.5 h) [37,38,41]. Parameters for sulfidity, AA and L : W ratio were based on the oven-dry weight of reed chips (w/w). After each pulping batch, the cooked material was unloaded, thoroughly washed with room temperature water until neutral, and then fiberized using a disintegrator at 75,000 rpm. The fiberized material was passed through a filter of 0.16 mm pore size and subsequently oven-dried at 60°C until reaching a constant weight.

Pulp cooking effectively dissolves up to approximately 90% of the lignin without significantly degrading the cellulose fibre. However, additional delignification through the bleaching process is necessary to produce high-quality cellulose. The bleaching sequence and the number of stages are primarily designed based on the desired brightness levels and intended paper grades [36,37,43–45]. A simple two-stage bleaching sequence (HP) was applied to the obtained cellulose at room temperature. In the first stage (H), sodium hypochlorite (NaOCl) was used as an inexpensive and efficient delignifying agent. A mixture of 10 g of obtained cellulose and 50 ml of NaOCl (2%) was continuously stirred in a 250 ml glass beaker for 3 h. In the second bleaching stage (P), the resulting cellulose underwent H₂O₂ treatment (5%) for 2.5 h. H₂O₂ was employed because it is eco-friendly, promotes brightness, prevents brightness loss over time and preserves the strength and integrity of cellulose [45–47]. After each bleaching stage, the material was thoroughly washed with hot water at 80°C and then dried until reaching a constant weight. This purified cellulose is referred to as isolated cellulose (RC). After treating RC with nitric acid and ethanol, following the Cross and Bevan method, the RC content, based on the final obtained RC weight (m1), was determined by the following equation [48]:


(2.1) $$RC\;\;content\;\left(\;\%\right)=\frac{m_{1}}{0.5}\times 100.$$

### 2.3. Fabrication of RCA_MTMS

First, RC (1%, wt) was dispersed into a 5% NaOH/PEG aqueous solution (1 : 4 volume ratio, v/v) by sonicating for 7 min using a 20 kHz Sonic device at 70% power at room temperature to form a homogeneous solution. Next, the solution was poured into a plastic mold, sealed and put in a Biomedical Freezer MDF-U5312 at −30°C for 20 h to allow gelation. The obtained gel was transferred to a glass beaker containing a 1% HCl solution, which was replaced several times until the gel became neutral. Distilled water was used for thorough rinsing until a neutral pH was achieved. Afterwards, the gel was immersed in EtOH and then placed in a vacuum drying cabinet (LaboGene Touch 1110-4) to shape its structure for 48 h at −50°C [14,49]. Upon completion of freeze-drying, reed-based cellulose aerogel was generated, followed by modification using MTMS by a chemical vapour deposition method. A sealed glass container, including 1 g of RCA and a small open glass vial containing 300 μl of MTMS, were placed in a drying cabinet at 70°C for 2 h to coat MTMS onto the RCA surface. The resulting RCA_MTMS was placed in a vacuum oven to remove any excess MTMS [5,28].

### 2.4. Characterizations

RC’s brightness and ash content were determined following the procedures outlined in TAPPI T218 (Spectrophotometer CM-5) and TAPPI T 413, respectively.

The surface morphology of RC, RCA and RCA_MTMS was observed using a SEM (Hitachi S-4800, Japan). The chemical composition of RC, RCA and RCA_MTMS was recorded using FTIR spectra obtained with the KBr disk method on a Bruker Tensor instrument (Germany). The analysis covered the wavenumber range of 4000–500 cm⁻¹ with a resolution of 4 cm⁻¹.

The pore volume and pore size distribution of RCA and RCA_MTMS were determined from nitrogen adsorption–desorption isotherms using Quantachrome Novawin and were estimated by the BET and the Barrett–Joyner–Halenda methods.

WAC of RCA_MTMS was measured at room temperature and ambient relative humidity using an OCA 20 contact angle meter (Dataphysics, Germany) to investigate the water repellency of the RCA_MTMS.

Thermogravimetric analysis was performed to compare the degradation characteristics of RC, RCA, and RCA_MTMS. The thermal stability of each sample was determined using a thermogravimetric analyser (LABSYS evo) at a heating rate of 10°C/min in the temperature range of 20 to 600°C.

### 2.5. Crude oil absorption experiments

The crude oil adsorption capacity of RCA_MTMS was evaluated following the procedure of the ASTM F726-06 method with slight modifications [5]. The dimensions of the 0.1 g sample of RCA_MTMS used for the absorption test were 45 mm (diameter) × 11 mm (thickness). The RCA_MTMS sample was placed in 300 ml of crude oil (Ruby crude oil with density at 15°C: 0.84 g ml^−1^ and viscosity at 50°C: 8.61 cST) for 30 min. Subsequently, the wet sample of RCA_MTMS was lifted vertically using a wire net and allowed to drain for 1 min by suspending the net over a beaker. Next, its weight and dimensions were determined, followed by hand-squeezing, and finally, it was weighed again. The oil adsorption capacity of RCA_MTMS was calculated by the following equation:


(2.2) $$AC\left(g\;g{\;}^{-1}\right)\;=\frac{m_{2}-m_{1}}{m_{1}},$$

where AC (g g^−1^) represents the crude oil absorption capacity of RCA_MTMS after *t* time (second).

*m*_1_ (g) the weight of the RCA_MTMS before absorption.

*m*_2_ (g) is the weight of the RCA_MTMS after absorption.

Additionally, the oil absorption capacity from the water surface, as a practical performance of RCA_MTMS, was examined. A 0.722 g sample of RCA_MTMS was placed on an oil layer floating atop the water in a Petri dish. This layer was achieved by slowly pouring 5 ml of crude oil onto 40 ml of water.

## 3. Results and discussion

### 3.1. Effect of sulfidity on RC content

**Figure 1.**
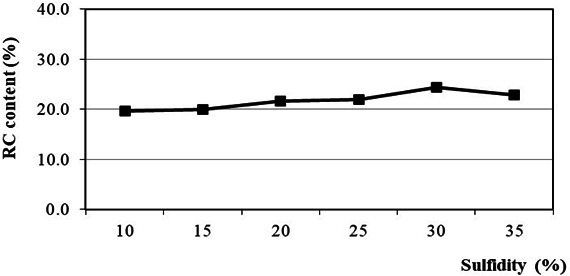
Effect of sulfidity on RC content.

The effect of sulfidity on reed-isolated cellulose (RC) content is shown in figure 1.

It is observed that the highest RC content is 24.0% at a sulfidity of 30%. Increasing the sulfidity charge leads to a higher delignification level due to the higher concentration formation of [HS^-^] ions formed from the dissociation of Na₂S. This, in turn, accelerates the delignification and degradation of cellulose, resulting in an increase in isolated cellulose content. However, an excessively high sulfidity charge (above 30%) leads to a decreasing trend in isolated cellulose content. This can be attributed to the loss of fine cellulose fibres, which tend to adhere to larger lignin molecules during the washing step. Moreover, excessively high sulfidity levels may lead to increased emissions of reduced sulfur compounds, leading to higher corrosion rates in equipment systems [36,38,41,43].

### 3.2. Effect of AA on RC content

**Figure 2.**
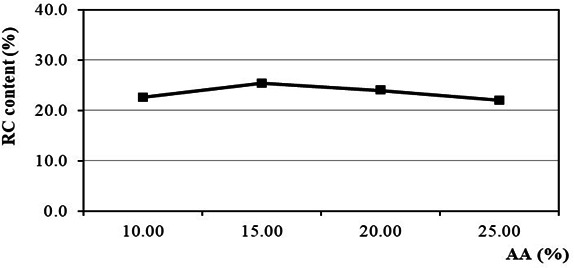
Effect of AA on RC content.

AA includes NaOH and Na_2_S, which are active agents in Kraft pulping. AA supplies [OH^−^] and [HS^−^] ions for the delignification and degradation of carbohydrates such as cellulose and hemicellulose. The effect of AA on RC content is shown in figure 2. The results indicate that the highest RC content is 25.4% at an AA of 15%. Increasing the AA charge above 15% leads to a higher concentration of [OH^−^] and [HS^−^], thus accelerating the degradation and hydrolysis of cellulose. As a result, the increased fine cellulose fibres, which are easily eliminated during the washing and screening process, contribute to the decrease in RC content. Notably, AA charge influences both [OH^−^] and [HS^−^] ion concentration, while sulfidity charge primarily affects [HS^−^] ion concentration. Overall, RC content is more significantly and sensitively affected by AA than by sulfidity. These results are consistent with previous studies [36–38,41].

### 3.3. Effect of L : W ratio on RC content

**Figure 3.**
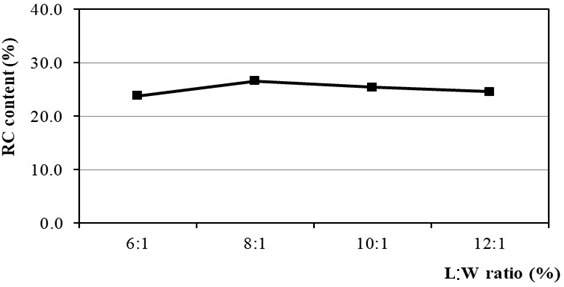
Effect of L : W ratio on RC content.

The L : W ratio is an important factor affecting Kraft pulping performance because it produces strong alkaline media for pulping and solubilizing lignin, hemicellulose and cellulose. To ensure effective impregnation of feedstock chips with active chemicals, an appropriate volume of pulping liquor must be calculated to provide a sufficient amount of ions, specifically [OH^−^] and [HS^−^] ions for the cooking reactions through the dissociation of NaOH and Na_2_S. In contrast, insufficient liquor volume may result in uncooked chips, leading to increased rejects and a decrease in RC content. Figure 3 illustrates the positive effect of the L : W ratio on RC content, showing that the highest RC content is 26.6% at an L : W ratio of 8 : 1. However, increasing the L : W ratio above 8 : 1 reduces the concentration of both [OH^−^] and [HS^−^] ions, resulting in decreased delignification and degradation of cellulose and hemicellulose, followed by a decrease in RC content. Moreover, an excessive L : W ratio also increases costs related to subsequent evaporation in the recovery process [36,50].

### 3.4. Effect of cooking time on RC content

**Figure 4.**
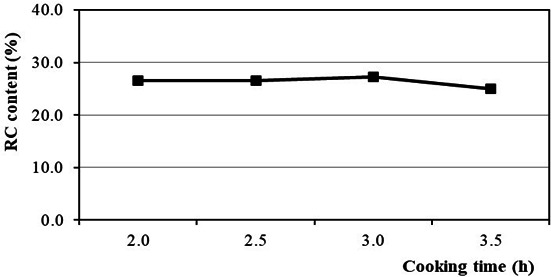
Effect of cooking time on RC content.

The effect of cooking time on RC content is shown in figure 4. The results indicate that the highest RC content is 27.20% at a cooking time of 3 h. During this period, the pulping liquor effectively penetrates the reed chip structure, efficiently promoting pulping chemical reactions. However, increasing the cooking time to more than 3 h in a strongly alkaline medium and at a high temperature accelerates lignin recondensation. Unfortunately, this complicates the isolation process of cellulose and leads to a decrease in RC content. Furthermore, prolonged cooking time leads to significant cellulose degradation. As a consequence, pulping products break down into fine fibres that are easily dissolved during the washing and screening process and cannot be recovered [50].

### 3.5. Characterizations

Cellulose isolated from reed (RC) exhibits high brightness (80% ISO) and low ash content (1%). Brightness closely correlates with the residual lignin content of the isolated cellulose; a high brightness of 80% ISO indicates that lignin is almost completely removed. These findings align with the research of Ates *et al*. [32] and Shatolov *et al*. [42,45,51,52]. It is notable that the RC fabrication process is simpler and does not require organic solvents for pulping and bleaching. The successful application of the conventional Kraft method in the pulping industry allows efficient large-scale RC production and these characteristics of brightness and ash content contribute to the favourable physical–chemical properties of the aerogel prepared from RC.

**Figure 5.**
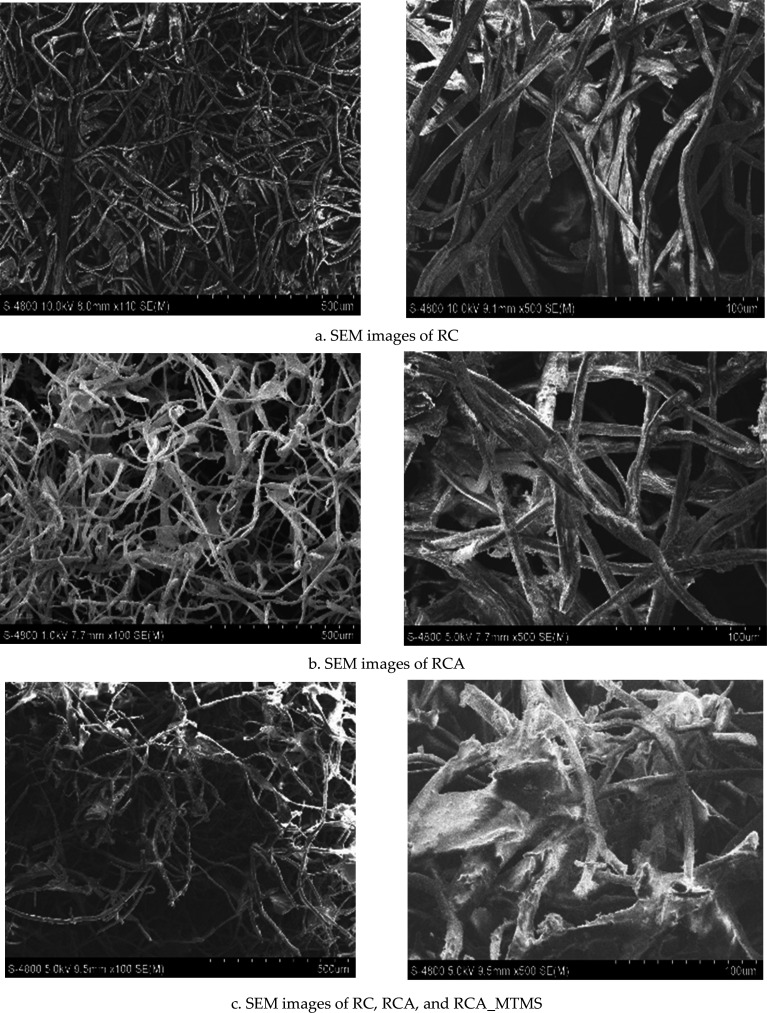
SEM images of the RC, RCA and RCA_MTMS.

As shown in figure 5, SEM images of RC, RCA and RCA_MTMS all exhibit relatively homogeneous surfaces with interconnected, uniform, long fibres forming a highly porous three-dimensional structure.

Interestingly, there are no observable signs of condensed lignin residue precipitating on the RC surface. However, noticeable morphological changes in both RCA and RCA_MTMS are comparable to RC. They appear to exhibit smaller interconnected fibres and a higher porosity structure. This can be attributed to the freeze-drying technique. Rapid sublimation of ice particles from the initial RC structure increases cross-linking among cellulose chains, preserving the porous structure [27,53,54]. Consequently, this process leads to the formation of an aerogel characterized by low density, high porosity, large specific surface area and a more uniform and smooth surface structure. It is clear that after MTMS modification, the cellulose fibres of RCA_MTMS are covered with a thin membrane. Compared to RCA, its pores are smaller, and its three-dimensional structure is tighter. This is due to the grafting of siloxane groups from MTMS with the hydroxyl (−OH) groups of RCA, resulting in a denser network, smaller pores and a rougher surface [55,56]. Despite these changes, the highly porous three-dimensional network of RCA_MTMS is still well-preserved. The interconnected three-dimensional network of pores allows the rapid and deep movement of oil into the aerogel, enhancing the absorption rate and facilitating the distribution of the absorbed oil throughout the aerogel [3,20].

**Figure 6.**
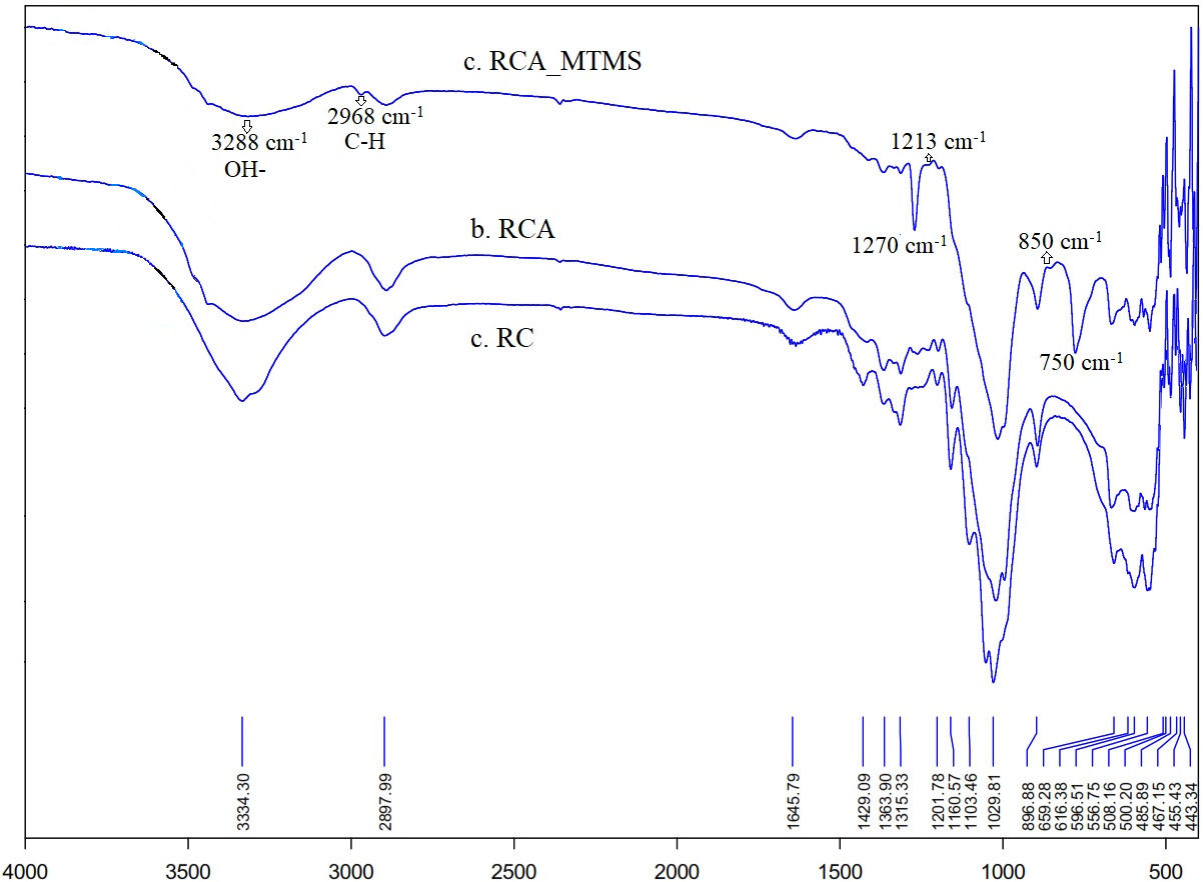
FTIR spectra of the RC, RCA and RCA_MTMS.

The change of chemical structures of RC, RCA and RCA_MTMS was studied using FTIR spectra presented in figure 6. Consistent with previous studies, all spectra of RC, RCA and RCA_MTMS exhibit characteristic peaks associated with cellulose [33,44,47,57–59]. These characteristic peaks are present in both FTIR spectra of RCA and RCA_MTMS, indicating that the processes of freeze-drying and MTMS modification do not change the main chemical functional groups of cellulose. Notably, peaks at 3340, 1750, 1663, 1600, 1510 and 1250 cm^−1^ are absent. Based on research by Balaji and Narajanan, along with other reports, this confirms that lignin and hemicellulose are nearly completely removed [33,44,47,57–59]. Furthermore, a sharp peak at 1213 cm^−1^ is observed in all three spectra, correlating with an increase in the purity of cellulose [44,57]. Remarkably, after hydrophobic modification, the FTIR spectra of RCA_MTMS display distinct and intense peaks attributed to the stretching vibrations of C–H bonds of Si–CH₃ groups at 2968 and 750 cm^−1^, respectively, as well as Si–C and Si–O–Si bond at 1270 and 850 cm^−1^, respectively. The noticeable decrease in O–H stretching intensity near 3334 and 1645 cm^−1^ indicates a successful silanization reaction between the abundant hydroxyl groups of cellulose and MTMS, forming covalent Si–O–Si bonds on the RCA_MTMS surface. The dramatic reduction in the number of hydroxyl groups and introduction of hydrophobic groups –O–Si–(CH_3_)_3_ groups from MTMS on RCA_MTMS surface, make it water repellent and oleophilic. These properties are particularly effective in oil spill clean-up and oil-water separation. These FTIR results are consistent with the slightly altered surface of RCA_MTMS observed in the SEM results mentioned above, confirming that the fabrication process of RCA_MTMS preserves the chemical composition of reed-isolated cellulose.

**Figure 7.**
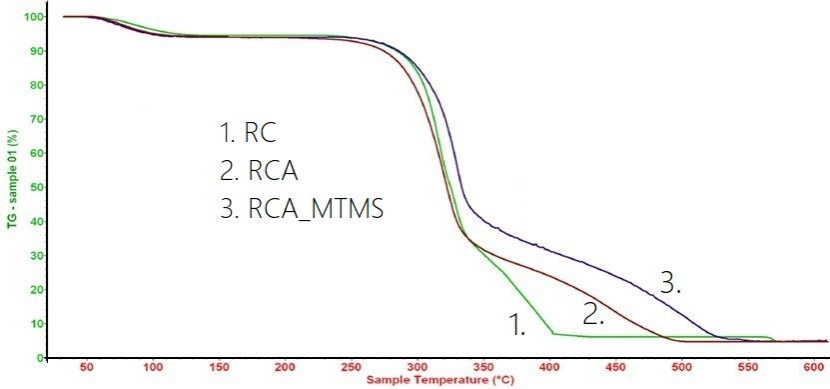
TGA curves of the RC, RCA and RCA_MTMS.

Figure 7 presents the TGA thermogram of RC, RCA and RCA_MTMS. Notably, all three materials exhibit similar thermal behaviours and three distinct weight loss stages. Initially, slight weight losses are observed below 140°C for RC, RCA and RCA_MTMS, corresponding to 5.4, 5.1 and 3.7%, respectively. These weight losses result from the evaporation of adsorbed water and volatile chemicals [57,58]. RC and RCA, being hydrophilic, exhibit greater weight loss from dehydration during this stage. The most significant weight loss occurs between 250 and 400°C, with values of 67.2, 61.9 and 39.7% for RC, RCA and RCA_MTMS, respectively. This weight loss can be attributed to cellulose glycosyl unit degradation processes, including dehydration, decarboxylation, depolymerization and decomposition, accompanied by char formation [13,58]. In the final weight loss stage, occurring between 350 and 550°C, RC, RCA and RCA_MTMS exhibit weight losses of 21, 21 and 17%, respectively. This stage involves the oxidation of cellulose, degradation of remaining lignin and the breakdown of –Si–O–C, –Si–O–Si– groups [60]. After this stage, the mass of all material samples remains relatively constant due to the presence of stable inorganic residues. Remarkably, RCA_MTMS consistently exhibits significantly lower weight losses at higher temperature, indicating its superior thermal stability compared to RC and RCA. Moreover, the highest residual mass of RCA_MTMS at the end of the TGA graph (typically above 600°C) also indicates the presence of thermally stable silica.

**Figure 8.**
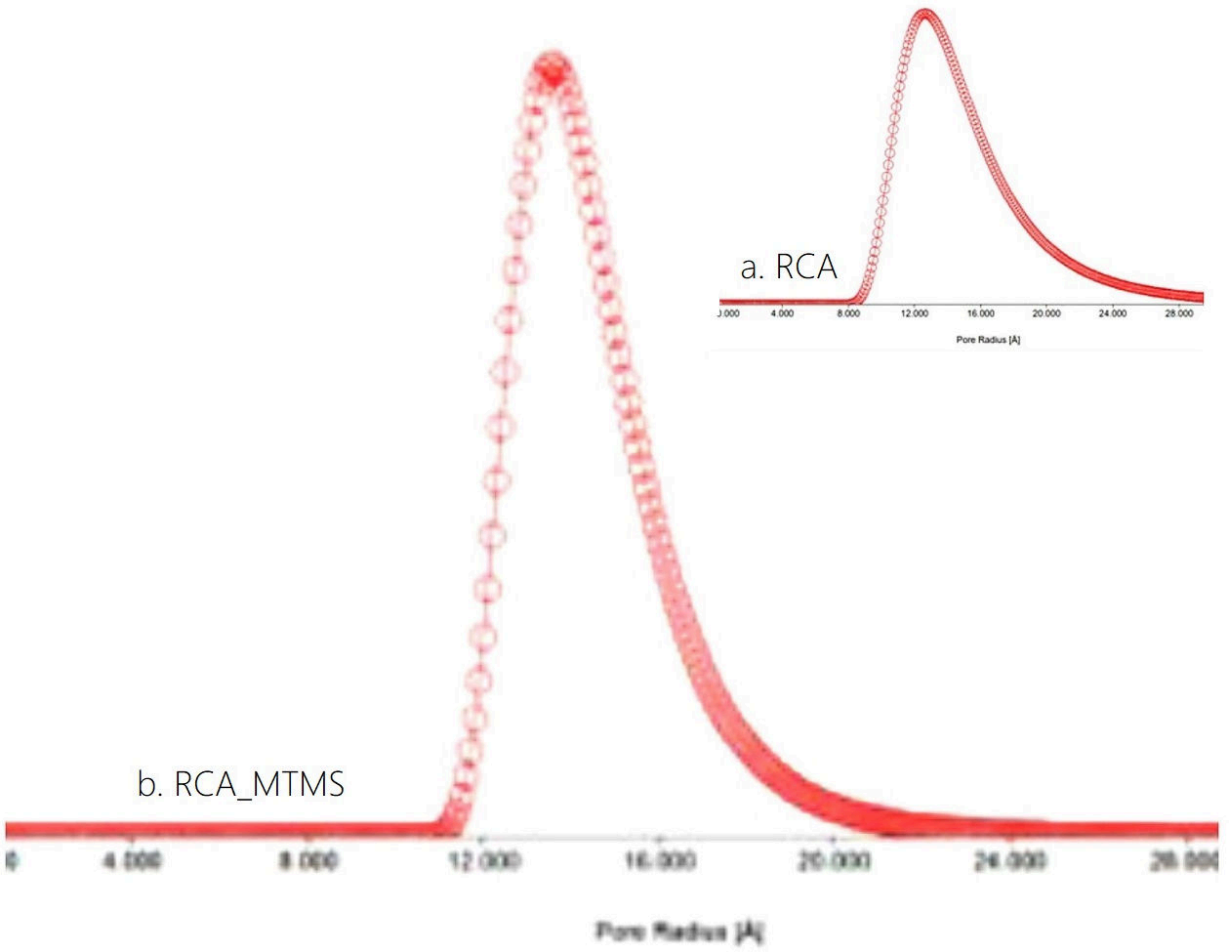
The pore size distribution of the RCA, RCA_MTMS.

The analysis results indicate that RCA_MTMS has a low density of 0.04 g cm^−3^ and a high porosity of 96%. These values are consistent and comparable to those of previous studies using various feedstocks such as paper waste (0.04 g cm^−3^ and 97.3%) [5], rice straw-PVA (0.05−0.06 g cm^−3^ and 96.6%) [13], office paper waste with polyester resin (0.032−0.051 g cm^−3^ and 98–96%, respectively) [61], reed-based nanofibril cellulose (0.49 g cm^−3^), and bamboo-based nanofibril cellulose (0.57−1.095 g cm^−3^) [55]. As shown in figure 8, the pore volume of RCA_MTMS is 0.005 cc g^−1^, which is lower than RCA’s value of 0.012 cc g^−1^.

RCA_MTMS exhibits a narrower pore size distribution (ranging from 1.2 to 2.2 nm) with an average size of 1.36 nm, classified as micropores (<2 nm). In contrast, RCA’s pore size ranges from 0.8 to 28 nm with an average size of 1.26 nm. The hierarchical pore structure, including both micropores and mesopores, forms a network that effectively traps oil molecules and enhances capillary action, thereby drawing oil into the aerogel more efficiently [2,49,56]. Additionally, the rougher surface of RCA_MTMS can also increase the adsorption of oil due to enhanced van der Waals forces and hydrophobic interactions, promoting better adhesion between the oil and the aerogel surface. The hydrophobic, highly porous three-dimensional structure of RCA_MTMS not only allows oil to be entirely absorbed but also can then be recovered after clean-up [2,3,20,55].

These results suggest that the MTMS modification of RCA not only enhances the hydrophobic and oleophilic properties of RCA but also reconstructs a uniform pore size distribution, increasing the average pore size for RCA_MTMS. These properties efficiently support oil absorption. TGA observations confirm the SEM images discussed earlier.

**Figure 9.**
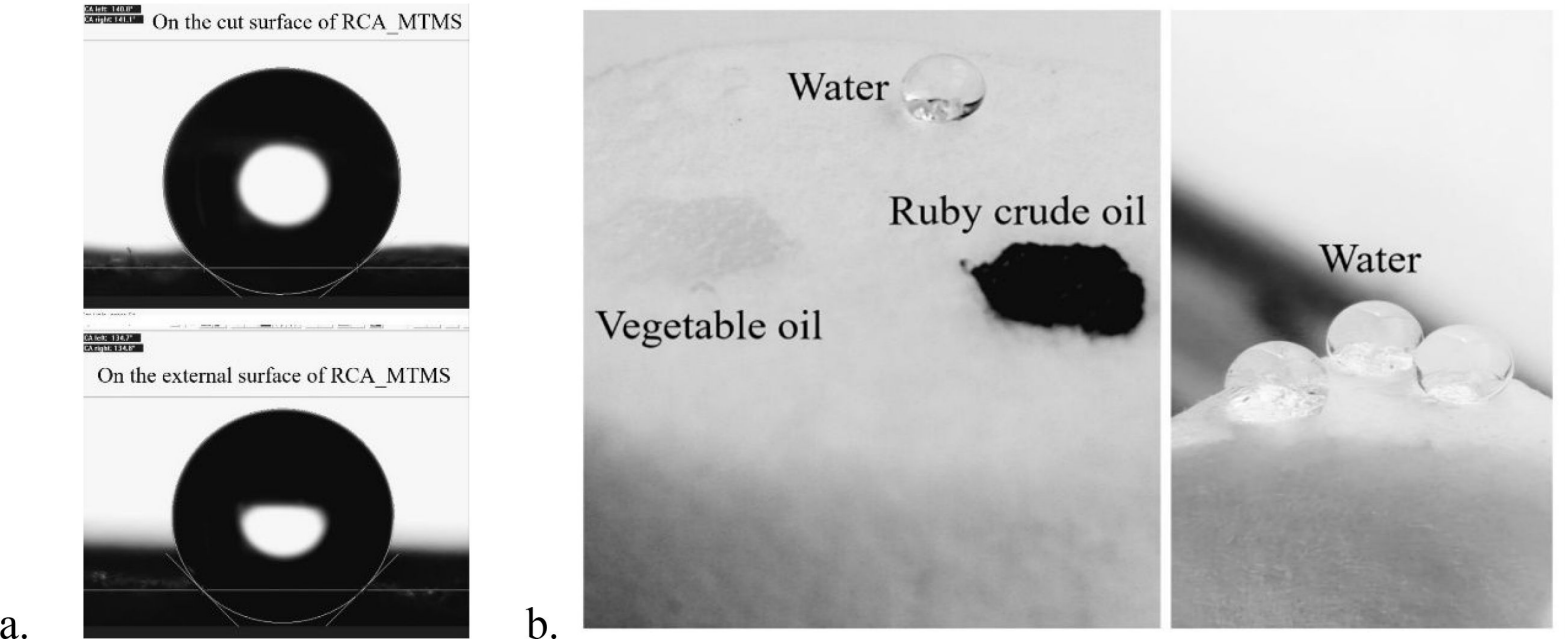
The hydrophobic property of RCA_MTMS.

To evaluate the effect of the MTMS coating on hydrophobicity, a WCA measurement was carried out for both the external surface and the cut surface of RCA_MTMS. As shown in figure 9*a*, the WCA of RCA’s external surface and the cut surface are 141^o^ and 138^o^, respectively. This confirms that the entire porous structure of RCA_MTMS exhibits high hydrophobic properties. Furthermore, figure 9*b* clearly demonstrates that RCA_MTMS can stably hold water droplets on its surface during testing, while drops of Ruby crude oil and vegetable oil are absorbed immediately and completely. These values are comparable to those reported in previous research on MTMS-coated cellulose aerogels such as a paper waste aerogel (145^o^) [5], nanofibril cellulose aerogel (128^o^) [12], reed-derived cellulose nanofibril aerogel (151−155^o^ for various liquids) [33], bamboo-derived cellulose nanofibril aerogel (132−138^o^) [55], office paper waste with polyester resin (138^o^) [61] and rice straw-cationic starch aerogel (137^o^) [62]. This consistency observed in the results confirms the successful MTMS coating onto RCA, significantly enhancing both water repellency and the oil adsorption capacity.

### 3.6. Oil adsorption performance

**Figure 10.**
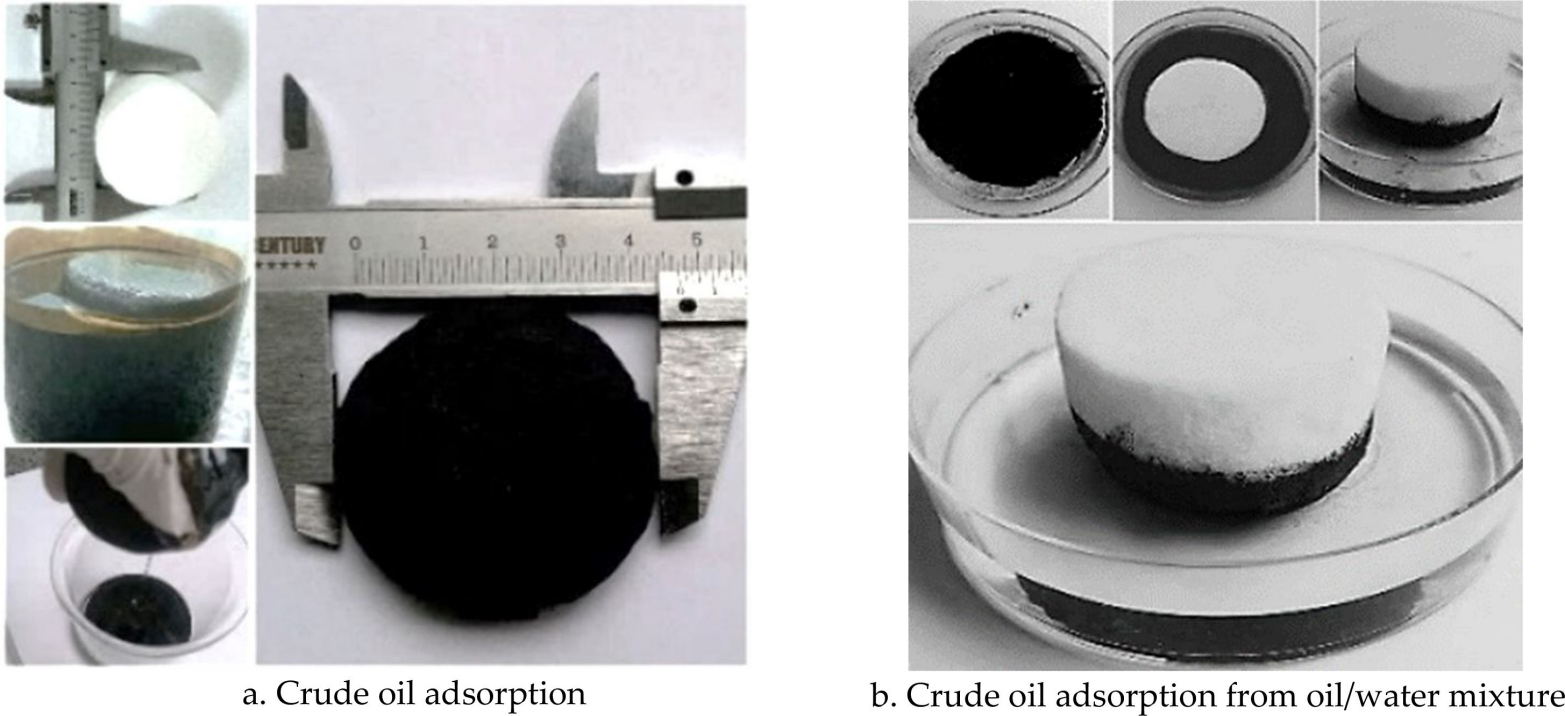
The crude oil absorption test of RCA_MTMS.

Figure 10 highlights the crude oil absorption capacity of RCA_MTMS. Interestingly, as shown in figure 10*a*, even after the absorption of crude oil and subsequent manual squeezing, RCA_MTMS maintained its original form without any notable deformation or size change. RCA_MTMS exhibits an efficient adsorption capacity of Ruby crude oil, approximately 35.5 times its dry weight in the first cycle after 5 min. This result is higher compared to that of recently developed cellulose aerogels under similar conditions for crude oil absorption using various feedstocks such as paper waste (18.4 g g^−1^) [5], rice straw (12−13 g g^−1^) [13], waste wheat straw (6−20 g g^−1^) [11,49] and office paper waste with polyester resin (29.67 ± 0.39 g g^−1^) [61] but aligns closely with cotton linter (34.5 g g^−1^) [8]. Moreover, RCA_MTMS’s absorption capacity is significantly lower than that of nanofibrillated cellulose aerogel from natural reed powder, as reported by Yue Jiao *et al*. (82.43 g g^−1^ for machine oil) [33]. Despite its promising potential for oil absorption, this material faces challenges related to the cost of NC fibrillation and the recovery of organic solvents, such as benzene and *tert*-butyl alcohol.

The crude oil adsorption capacity of RCA_MTMS is higher than the biodiesel absorption capacities of the cardboard-based aerogel (26.1 g g^−1^) and those of the office paper-based sample (29.9 g g^−1^) [63]. This difference can primarily be attributed to the additives used to enhance the properties and performance of paper in the industrial production, such as fillers, wet strength agents, sizing agents. Cellulose aerogel from purified MCC exhibits a machine oil absorption capacity of 12 g g^−1^ [56]. A similar observation was reported in a previous study, indicating that the oil adsorption capacity of MCC aerogel is lower than that of dissolving pulp cellulose [64]. Consequently, the oil absorption performance of RCA_MTMS is highly competitive compared to cellulose aerogels derived from other plant sources. Moreover, it is noteworthy that the fabrication process of reed-based cellulose aerogels is simpler, greener, and more cost-effective. Importantly, this process can be scaled up for mass production by utilizing technology and equipment from the pulp industry to isolate cellulose from reed. Furthermore, RCA_MTMS effectively separates crude oil from a mixture of Ruby crude oil and water, as shown in figure 10*b*. RCA_MTMS floats on the mixture and selectively and rapidly absorbs nearly all the crude oil, leaving mostly water below, after approximately 3 min. This indicates that the MTMS hydrophobic coating occurs not only on the surface of RCA but also throughout its internal structure, consistent with the above-discussed WCA results. Once the absorbed oil is removed, RCA_MTMS can be reused for subsequent absorption cycles. The reusability and absorption efficiency of RCA_MTMS for various oils will be discussed in our upcoming studies. The results above indicate that the developed RCA_MTMS has efficient adsorption and separation capabilities, highlighting its significant potential as a material for oil spill clean-up.

## 4. Conclusion

Hydrophobic cellulose aerogels, fabricated from reed as a raw material, were successfully developed using the freeze-drying method and an eco-friendly NaOH/PEG aqueous solvent system, followed by MTMS modification. The highest content cellulose, obtained after mild Kraft pulping and bleaching, was 27.2% with high brightness (80% ISO) and low ash content (1%) under specific conditions: 20% AA, 30% sulfidity, a sustained cooking temperature of 165°C for 3 h, and a liquor-to-reed ratio of 8 : 1. The MTMS-coated cellulose aerogel exhibited extraordinary characteristics, including a low density of 0.04 g cm^−3^, high porosity of 96%, high hydrophobicity with a WCA of 141°, and an impressive oil adsorption capacity of 35 g g^−1^ [65]. The fabrication process is simple, cost-effective and eco-friendly, using reed as an abundant, low-cost, biodegradable and renewable raw material. Importantly, this study leveraged conventional existing technology in pulping and bleaching factories, enabling environmentally friendly, efficient and straightforward isolation of reed cellulose. These results significantly contribute to scaling up cellulose aerogel production. Overall, this interdisciplinary research provides a framework for further investigating reed as an alternative material for commercial aerogel production, with potential applications in sustainable solutions for oil spill clean-up and various environmental contexts.

## Data Availability

The datasets supporting this article have been uploaded as part of the electronic supplementary material (ESI) [65]. Supplementary material is available online [66].
